# Sports tourism motivation and tourist satisfaction in resort Ski tourism: A distal mediation model of tourist expectation and experience quality

**DOI:** 10.1371/journal.pone.0342176

**Published:** 2026-07-06

**Authors:** Mingliang Zhang, Chen Zhang, Shuyan Li, Tao Li, Yankun Han

**Affiliations:** 1 College of Physical Education, Jilin Normal University, Siping, Jilin Province, China; 2 Archives Center, Southwest Medical University, Luzhou, Sichuan Province, China; 3 Teaching Affairs Office, Jilin Normal University, Siping, Jilin Province, China; 4 School of Physical Education, Southwest Medical University, Luzhou, Sichuan Province, China; 5 College of Physical Education, Beihua University, Jilin, Jilin Province, China; University of Gdansk Faculty of Economics: Uniwersytet Gdanski Wydzial Ekonomiczny, POLAND

## Abstract

The popularity of leisure skiing culture presents a paradox with declining visitor satisfaction, and this study aimed to elucidate the mechanisms influencing tourist satisfaction in leisure ski tourism through a distal mediation model. Data were collected from 302 valid samples at Changbaishan Wanda International Ski Resort within the Changbaishan National Reserve in Jilin Province, Northeast China, using mixed offline-online methods. Structural equation modeling was conducted via IBM SPSS Statistics and AMOS 26.0, utilizing maximum likelihood estimation combined with Bollen-Stine bootstrap for hypothesis testing. While direct effects confirmed all hypotheses except hypothesis 5, results further revealed significant positive mediating effects: sport tourism motivation exhibited a distal mediation effect on tourist satisfaction through tourist expectation and experience quality and an independent mediation effect through experience quality alone. These findings collectively demonstrate that tourist expectation and experience quality mediate the relationship between motivation and satisfaction in leisure skiing contexts.

## Introduction

Ski tourism serves as a predominant activity within winter tourism offerings [1], experiencing rapid global expansion in recent years. This industry can be categorized into three primary sectors: competitive skiing, adventure skiing, and leisure skiing [2]. Competitive skiing primarily targets professional athletes and elite enthusiasts, emphasizing technical challenges and performance outcomes. Adventure skiing focuses on extreme exploration in remote terrains, prioritizing thrill-seeking and adventurous experiences. In contrast, leisure skiing emphasizes recreational value, relaxation, and family participation, catering to diverse age groups and skill levels [3].

With the growing popularity of ski culture and continuous improvements in ski infrastructure, leisure skiing has emerged as a significant component of mass tourism, driven by visitors’ desires to engage in physical activities within alpine natural environments [4]. Consequently, leisure ski tourism not only provides opportunities for winter sport participation but also functions as a critical economic pillar in many regions [5]. However, the proliferation of leisure ski tourists has concurrently led to environmental degradation in ski destinations, alongside emerging issues such as declining visitor satisfaction and low revisit rates [6]. Negative media reports from specific ski resorts have further exacerbated rising negative sentiment among leisure skiers, significantly impairing overall tourism experiences and satisfaction levels.

In fact, the ability of tourist attractions to attract visitors and cultivate positive reputations hinges fundamentally on satisfaction levels [7]. Therefore, tourist satisfaction has emerged as the core determinant of the sports tourism market’s sustainable development and an important predictor of revisit intention [1]. Originating from Cardozo [8] seminal work, customer satisfaction theory posits that fulfillment positively influences repurchase behavior. Subsequent research reveals this construct’s inherent complexity, encompassing not only physiological factors but also affective and cognitive-psychological dynamics [9]. Meanwhile, as a factor within cognitive-psychological dynamics, motivation reflects the psychological manifestation of personal needs and thereby compels individuals to engage in specific tourism activities or visit particular destinations to satisfy their psychological needs [10]. Studies demonstrate that motivation significantly impacts tourist satisfaction [11], functioning as a synergistic interplay between demand and desire that generates affective inclination toward destination experiences [7]. Meanwhile, congruence between motivation and experiential outcomes amplifies satisfaction levels [12]. Within sports tourism, motivators typically include health pursuits, social connectivity, skill enhancement, and stress escape [13]. Academic consensus posits that activation of dual intrinsic “pull” motivations (destination appeal) and extrinsic “push” motivations (internal drives) simultaneously catalyzes sports tourism engagement and enhances satisfaction outcomes [14]. Empirical evidence demonstrates a significant positive correlation between sports tourism motivation and tourist satisfaction [15], with satisfaction levels showing marked enhancement when motivational needs are fulfilled [16]. While existing studies have identified partial advancements in elucidating the relationship between sports tourism motivation and satisfaction, critical gaps persist in understanding the mediating mechanisms [17]. Accordingly, this study proposes to construct a mediation effect model to systematically investigate how sports tourism motivation influences visitor satisfaction through the mediating roles of tourist expectations and perceived experience quality. This approach aims to expand the mediating explanatory mechanisms underlying the motivational-satisfaction dynamic in sports tourism contexts.

On one hand, according to Expectation Confirmation Theory, tourist satisfaction is theorized to emerge from the discrepancy between pre-trip expectations and post-experience outcomes [18]. Where actual experiences exceed expectations, satisfaction levels increase; conversely, discrepancies below anticipatory thresholds result in diminished satisfaction [19]. Within leisure ski tourism contexts, visitor expectations encompass multiple dimensions including ski facility quality, service delivery, safety protocols, and snow conditions [20], with empirical observations confirm that congruence between these expectations and experiential realities directly modulates satisfaction trajectories-a phenomenon particularly salient in ski resort tourism ecosystems. Consequently, expectation confirmation emerges as a mediating mechanism to explain satisfaction formation processes [12].

On the other hand, visitor satisfaction is predominantly determined by overall travel experiences [21], with high-quality experiential quality directly enhancing satisfaction levels [22]. Theoretically, therefore, when tourism activities deliver positive experiences and psychophysical gratification, visitors perceive elevated experiential quality. Higher experiential quality yields progressively stronger satisfaction impacts, thus amplifying overall visitor satisfaction. Consequently, leading tourists to develop a psychological state encompassing both objective service quality and subjective emotional experiences and affective responses [23]. Although some studies have demonstrated that experience quality can function as a mediating variable to clarify the relationship between expectations and satisfaction [7], in the context of leisure ski tourism, experience quality is influenced by a multitude of factors-such as ski resort infrastructure, instructor expertise, and weather conditions [24]. Thus, whether experience quality can serve as a mediating variable to explain the relationship between motivation, expectations, and satisfaction remains to be further validated by empirical research.

This study investigates the relationship between sports tourism motivation and visitor satisfaction within leisure ski tourism contexts, while incorporating expectations and experiential quality as mediating variables to construct a distal mediation model. Through this framework, the research aims to comprehensively elucidate the formation mechanisms of visitor satisfaction in leisure skiing and establish a theoretical foundation for optimizing management practices and service delivery in ski tourism destinations. Furthermore, this investigation contributes to expanding the theoretical framework within sports tourism studies, while offering novel perspectives and methodological advancements for future scholarly inquiries.

## Literature review

Drawing on Ryan and Deci [25] foundational typology of motivation, we categorize motivation within the leisure ski tourism context into two overarching types: intrinsic motivation and extrinsic motivation. Intrinsic motivation refers to an innate, natural disposition that cultivates human interest and develops capabilities [26]; it generates energy to fulfill the organism’s need to seek and achieve an optimal balance between competence, self-determination, and environmental harmony [27]. This motivation arises volitionally from internal intentions, driving behaviors without reliance on external contingencies like rewards or controls. When experiencing intrinsic motivation, individuals report feelings of interest [28], enjoyment, and fun [29]- outcomes linked to perceiving an internal locus of causality and enhanced competence and self-determination [30]. Extrinsic motivation, by definition, is the counterpart of intrinsic motivation—requiring external environmental intervention (e.g., rewards, pressure) to emerge [31]. Focusing on leisure ski tourism, within the dual domains of “leisure” and “tourism,” participants are typically initially motivated by the goal of enhancing physical and mental well-being. Consequently, the sports tourism motivation examined in this study specifically refers to intrinsic motivation in the context of leisure ski tourism.

Motivation and satisfaction constitute an interconnected pair of constructs across numerous research domains. For instance, studies in education [32], administrative sciences [33,34], public health [35], business [36], and tourism [37–39] have consistently explored this dynamic. A majority of findings indicate that enhancements in motivation significantly influence the expression of satisfaction. In administrative sciences, for example, Santos, Santos [33] demonstrated that employee motivation in organizations substantially affects job satisfaction. Concurrently, motivation acted as a partial mediating variable between internal communication and work satisfaction. Similarly, Bright [34] found that public service motivation among government employees directly predicted job satisfaction and indirectly enhanced it via perceived organizational privileges. Aljumah [36], surveying 300 business managers in Saudi Arabia, revealed that both intrinsic and extrinsic motivation significantly impacted job satisfaction—a relationship moderated by transactional leadership. Parallel evidence emerges from tourism research. Brogni, Tricárico [37], studying visitors and residents of Brazil’s sacred urban complexes, reported that motivations such as religious faith, cultural recreation, and mental relaxation significantly shaped satisfaction with these sites. Rejón-Guardia, Rialp-Criado [39], examining Spain’s cycling tourism market, found that contemplation, lifestyle alignment, and social interaction—motivation-related factors—substantially elevated tourist satisfaction and even increased revisit intention.

However, despite extensive exploration of travel motivations [38,40,41], a critical caveat persists: motivation is multifaceted and dynamic [42]. Consequently, the widely held assumption that motivation universally enhances satisfaction may harbor significant oversights. Empirical counterexamples underscore this nuance. Hava [32], in English language teaching, noted that not all motivational dimensions predict student satisfaction in English learning; specifically, attitude—subdimension of motivation—required long-term cultivation to boost satisfaction. Kitsios and Kamariotou [35] surveying healthcare workers in Greece’s public health system, found neither intrinsic nor extrinsic motivation significantly affected job satisfaction. Additionally, research on cycling tourism revealed diverse motivational drivers (e.g., motivational variability, event-specific features, and typology-based distinctions), further complicating the motivation-satisfaction link [39]. These findings highlight that the impact of motivation on satisfaction is context-dependent and bounded by specific conditions; without such boundaries, research risks descending into unfocused, uninformative generalizations. Against this backdrop, a critical research gap emerges: Does motivation influence satisfaction among participants in leisure ski tourism? The role of event-specific factors, as well as participant demographics, event characteristics, and regional attributes in moderating this relationship, remains unexplored. To address this gap, this study proposes: Hypothesis 1: Sports tourism motivation has a significant positive impact on tourist satisfaction.

As a critical factor in consumer psychology, tourist expectations exert a significant influence on consumer behavior. Parasuraman, Zeithaml [43] define tourist expectations as the anticipation of outcomes from a tourism experience, positing that they play a pivotal role in shaping overall travel experiences. Within the framework of tourist expectations, Rodríguez Del Bosque, San Martin [44] identify past experience, external communication, word-of-mouth communication, and destination image as key determinants. In fact, earlier research has already examined the antecedents of expectation formation, emphasizing not only these structural factors but also the role of personal motivation [45,46]. When stimulated by specific contexts, individuals anticipate favorable or unfavorable incentives based on the consequences of their actions—in other words, behavioral choices are driven by anticipated outcomes. From this perspective, the motivational process serves as a prerequisite for expectation formation [47]. According to the expectancy model, motivation represents the psychological drive to pursue a desired goal state [48]. Understanding this mechanism requires two conditions: first, the occurrence of the goal state must be predictable (i.e., an expectation must exist); second, the goal state must hold intrinsic value or appeal to the individual to function as a motivation [47]. Theoretically, therefore, the initiation of motivation predicts the generation of expectations.

Chunyang and Qu [49], in their survey of mainland Chinese tourists participating in Hong Kong tour groups, found that travel motivation directly influences tourist expectations and indirectly affects them through the mediating role of the cognitive image of a destination. This study highlights that travel motivation and destination image are key factors shaping tourist expectations and play crucial roles in their formation. However, motivation can also be conceptualized as evolving alongside the development of expectations [47]. Wong, Cheung [50], examining Hong Kong inbound and outbound tourists, further revealed that tourist expectations significantly influence their travel motivation and, through the mediating role of motivation, shape tourist attitudes. Based on the foregoing discussion, motivation and expectation may function as mutually influential variables across diverse research contexts. Nevertheless, prior studies have highlighted the need to examine boundary conditions—such as nationality and familiarity with the tourism destination—that shape expectation formation in future research [44]. Furthermore, Poria, Reichel [51] note that motivational research often operates under the common assumption that visiting historical sites is perceived as a recreational experience embedded in leisure activities in their investigation of heritage tourism.

Against this backdrop, critical questions emerge for leisure ski tourism: Does this taken-for-granted assumption hold in the context of leisure ski tourism? Does the motivation of tourists participating in leisure ski activities influence their expectations? Whether this relationship remains valid under specific boundary conditions warrants further empirical validation. Building on the above discussion and research hypotheses, this study proposes Hypothesis 2: Sports tourism motivation has a significant positive impact on tourist expectation.

The emergence of the experience economy [52] has catalyzed research on consumer experiences across service industries [53]. Experience itself represents an economic supply model capable of creating competitive advantage [54]. Thus, crafting and delivering “extraordinary” customer experiences has emerged as an effective strategy for economic organizations to enhance consumer satisfaction and loyalty while sustaining competitive differentiation [55]. Subsequent scholarship has explored customer experiences from diverse angles, including consumption experiences [55], service experience [56]. However, these seemingly interchangeable concepts fundamentally differ from experience quality. For instance, service quality is inherently a cognitive, transaction-oriented evaluation, whereas customers tend to assess experience quality through subjective and emotional lenses [57]. Empirical research in the wine tourism industry by Fernandes and Cruz [58] identified six dimensions of experience quality: Environment, Service Providers, Learning, Entertainment, Functional Benefits, and Trust. While this diverges somewhat from prior conceptualizations of tourism experience [59,60], these frameworks collectively underscore that experience quality reflects tourists’ psychological and social responses to destination performance [21].

Xia, Lee [38] found that tourist motivation significantly influences their service quality perceptions and satisfaction by investigating Chinese tourists in spa and wellness tourism. Conversely, Suhartanto, Agustina [61] reported that in Indonesian creative tourism motivation impacts experience quality only through push motivation. These conflicting findings highlight inconsistencies in research on motivation’s influence on experience quality—disparities attributed to, but not limited to, variations in tourism sectors and participant groups.

Notably, Suhartanto, Brien [62], in a creative tourism study, identified experience quality, perceived value, satisfaction, and motivation as antecedents of tourist loyalty, with experience quality significantly affecting motivation. This confirms that motivation and experience quality form a reciprocally influential pair. Nevertheless, the multifaceted nature of experience itself dictates variability across research domains [63]. As goods and services grow increasingly homogeneous, firms must deliver meaningful experiences to enhance product/service value [64]. Thus, unlike the service experiences provided by other tourism programs, whether the experience quality offered by leisure ski tourism can be influenced by motivation requires further empirical investigation. Building on the above discussion, this study proposes Hypothesis 3: Sports tourism motivation has a significant positive impact on experience quality.

Though consensus remains elusive regarding the conceptual definition of satisfaction among scholars, it is widely acknowledged that satisfaction encompasses not only cognitive and affective dimensions but also physiological and psychological dynamics [9]. In tourism, tourist satisfaction is a critical determinant of customer behavior and, consequently, the success of tourism destinations [65]. Thus, scholars have examined tourist satisfaction across diverse tourism programs [38,58,62]. Subsequently, researchers have defined tourist satisfaction as the interplay between pre-trip expectations and post-trip experiences, positing that satisfaction is achieved when tourists’ pre-trip expectations are met post-trip [21]. Over the past decade, tourism research on satisfaction has incorporated multiple variables, including motivation, service quality, satisfaction, and quality of life [38]; motivation, satisfaction, experience quality, value, and tourist loyalty [62]; experience quality, satisfaction, loyalty, and advocacy [58]; and motivation, satisfaction, and destination loyalty [12].

However, few studies have investigated and validated the relationships among motivation, tourist expectations, experience quality, and tourist satisfaction specifically within leisure ski tourism. As noted in prior discussions, the expression of tourist satisfaction is also constrained by boundary conditions. Thus, it remains unclear whether previously validated relationships—such as that between tourist expectations and satisfaction—hold in the unique context of leisure ski tourism, with its distinct participant groups and geographic settings. Further empirical research is warranted to address this gap. Regarding the relationship between motivation and satisfaction, this has been elaborated in the preceding review. This section now addresses the connections between experience quality and satisfaction, as well as between tourist expectations and satisfaction.

Visitor satisfaction is intrinsically linked to emotional experiences during tourism activities: positive emotions such as pleasure and surprise enhance satisfaction, whereas negative emotions trigger dissatisfaction [66]. Overall travel experiences primarily determine satisfaction levels [21], with high-quality experiences directly elevating satisfaction [22]. Perceived experience quality thus serves as a critical determinant of visitor satisfaction. In specific destinations, superior experiences yield heightened satisfaction [67]- a pattern particularly pronounced in sports tourism, where engagement depth and experiential intensity significantly influence satisfaction [60]. Fernandes and Cruz [58] in their wine tourism study, demonstrated that experience quality significantly impacts tourist satisfaction, loyalty, and advocacy, identifying six dimensions of experience quality: Environment, Service Providers, Learning, Entertainment, Functional Benefits, and Trust. González-Rodríguez, Domínguez-Quintero [68] further revealed that experience quality not only directly affects satisfaction but also indirectly influences it through mediating variables such as emotions and perceived value by examining historical heritage tourism.

However, Suhartanto, Brien [62], in a creative tourism study, found that while experience quality is a key determinant of tourist loyalty, it does not significantly affect satisfaction within their variable framework. These findings highlight variability in the impact of experience quality on satisfaction across studies—a discrepancy attributed to differences in how tourists perceive experience dimensions across tourism programs, an inevitable consequence of varying project characteristics. Consequently, the specific influence of experience quality on satisfaction in leisure ski tourism remains unexplored. Building on this gap, we propose: Hypothesis 4: Experience quality has a significant positive impact on tourist satisfaction.

Expectations are widely regarded as benchmarks consumers use to evaluate satisfaction and assess product/service performance. Rooted in Expectation Confirmation Theory, satisfaction stems from the congruence between pre-trip expectations and post-consumption experiences [69]. Positive confirmation—where experiential outcomes surpass expectations—enhances satisfaction, whereas negative disconfirmation diminishes it [65]. High expectations generally yield elevated satisfaction when outcomes meet or exceed anticipatory thresholds [70]. Wei, Zhao [71] further validated this relationship, demonstrating that the expectation-satisfaction linkage in ski resorts is mediated by perceived service quality. Theoretically, narrower gaps between expectations and experiences amplify perceived quality, thereby strengthening satisfaction. Luo and Ye [72] investigated visitors to the Macao Museum and found that tourist expectations significantly positively influence satisfaction; additionally, both expectations and satisfaction act as mediators in the relationship between generativity and museum visit intention. However, Minh, Rahman [73] did not confirm a significant direct impact of tourist expectations on satisfaction, positing that experiential factors are more critical via surveying international and domestic visitors to Vietnam. These findings highlight inconsistencies in research on the expectation-satisfaction relationship within tourism.

Theoretically, tourist expectations encompass multiple dimensions [44–46]. Given that contextual factors and group characteristics inevitably moderate these dimensions, the influence of expectations on satisfaction may vary across tourism contexts. Thus, whether tourist expectations significantly affect satisfaction in leisure ski tourism—consistent with prior findings—remains empirically unresolved. Building on this gap, we propose: Hypothesis 5: Tourist expectation has a significant positive impact on tourist satisfaction.

Tourist expectations—defined as the anticipatory outcomes of tourism encounters-play a pivotal role in shaping overall travel experiences [43]. Discrepancies between sport tourists’ expectations and their actual experiences directly influence perceived service quality. These expectations are formed through prior knowledge, marketing communication, and individual motivations [45], serving as antecedent variables that determine satisfaction levels by mediating perceptions of destination quality [74]. Oliver [69] posits that tourist expectations and actual experiences constitute the foundational components of tourism quality: when actual perceptions exceed expectations, a positive discrepancy emerges to enhance perceived quality; conversely, unmet expectations result in dissatisfaction.

Currently, few studies have focused on the relationship between tourist expectations and experience quality. Research on experience quality itself has largely been operationalized as service experience [56,75], with investigations into experience quality primarily centered on its relationships with variables such as satisfaction and loyalty [58], satisfaction and revisit intention [22], motivation and loyalty [62], and authenticity and satisfaction [76].Consequently, this study seeks to validate the relationship between these two variables through an examination of leisure ski tourism, aiming to provide actionable insights for future research. Building on this gap, we propose: Hypothesis 6: Tourist expectation has a significant positive impact on experience quality.

Recent scholarship has increasingly examined the indirect effects of motivation on satisfaction through mediating variables. For instance, Lee, Jeon [70] employed path analysis with Chinese outbound tourists to South Korea and demonstrated that tourist expectations mediate the relationship between motivation and satisfaction. Wang, Tran [77] further validated this mediating role of expectations. Additionally, motivation enhances experience quality, thereby indirectly influencing satisfaction [71]. Within tourism contexts, scholars define experience quality as the psychological and social reflections of tourist encounters. Notably, experience quality distinguishes itself from service quality: it encompasses not only in-trip service delivery but also interactions between tourists and destination elements (e.g., people, objects, environments). Thus, experience quality possesses a broader conceptual scope than service quality. Despite extensive research on tourism experience quality, many latent influencing factors remain unexplored [10]. Nevertheless, there is consensus that experience quality positively impacts satisfaction and revisit intentions [78]. For example, Chung and Petrick [79] showed that high-quality travel experiences significantly elevate satisfaction. Mediation occurs when a third variable transmits the effect of an independent variable on a dependent variable. Following participation in sports tourism, tourists evaluate destination quality based on actual experiences, comparing against their pre-trip psychological states, motivations, and expectations. Building on all the foregoing discussion, this study identifies tourist expectation and experience quality as two mediating variables and proposes the following hypotheses:

Hypothesis 7: Tourist expectation and experience quality exhibit a chain mediating effect between sports tourism motivation and tourist satisfaction;

Hypothesis 8: Tourist expectation plays a significant mediating role in the relationship between sports tourism motivation and tourist satisfaction;

Hypothesis 9: Experience quality plays a significant mediating role in the relationship between sports tourism motivation and tourist satisfaction.

## Research methods

### Data collection and participants

This study focuses on Changbaishan Wanda International Ski Resort, located in the Changbaishan National Reserve within Jilin Province, Northeast China. Situated at the core of the Changbai Mountain range, the resort represents one of the most representative premier ski destinations in Northeast Asia. Geographically advantaged, the resort is positioned 20 kilometers from Changbaishan Airport, ensuring accessibility that attracts substantial domestic and international skiing enthusiasts. The resort boasts 43 ski runs spanning approximately 30 kilometers, encompassing beginner, intermediate, and expert trails designed to accommodate diverse skill levels. Advanced snowmaking facilities and gondola systems ensure consistent skiing experiences and slope quality maintenance. Renowned for its superior service offerings, the resort features professional ski instruction teams, comprehensive amenities including equipment rental, dining, and lodging, alongside recreational attractions such as hot springs and ice theme parks. These elements collectively deliver holistic winter resort experiences. Changbaishan Wanda International Ski Resort’s competitive advantages stem from its unique natural terrain, internationally certified management standards, and diversified tourism products, solidifying its status as an optimal destination for leisure skiing tourism.

This study focuses on leisure skiers at Changbaishan Wanda International Ski Resort in Jilin Province, China. Data collection occurred during the 2025 January 1–February 25 period, encompassing peak skiing season to ensure sample diversity and representativeness. Participants were selected as regular participants in ski clubs or public skiing activities to guarantee substantive engagement with skiing tourism. A mixed-method survey design was implemented, combining online and offline data collection. Online questionnaires were distributed via QR codes, while on-site administration involved two leisure skiing experts and four trained student assistants who clarified questionnaire objectives to enhance response accuracy and completeness. A total of 330 questionnaires were distributed, yielding 302 valid responses, resulting in a valid response rate of 91.5%. The experiment was approved by the research ethics committee of Jilin Normal University. All subjects were verbally informed of the purpose and requirements of the research, provided written consent.

The power analysis conducted by MacCallum, Browne [80] determined that a minimum sample size of 133 is required for structural equation modeling analyses under the specified conditions: hypothesized root mean square error of approximation (RMSEA) = 0.08, degrees of freedom (df) = 98, significance level α = 0.05, and desired statistical power of 0.8. Notably, our study achieved an effective sample size of 302 participants, which exceeds the recommended threshold. Subsequent post-hoc calculations confirmed that the achieved statistical power for the current sample size (N = 302), maintaining all original parameters (RMSEA = 0.08, df = 98, α = 0.05), was exceptionally high at 0.996.

### Measurement scales

This study employed a survey method to measure variables, with questionnaire design grounded in literature reviews and established scales. Firstly, sports tourism motivation was assessed using Seo, Lewin [81] scale, encompassing eight dimensions (e.g., health pursuit, social needs, skill enhancement) across 28 items. Secondly, sport tourists’ expectation was measured via Sun, Xie [82] framework, including five dimensions (e.g., facility expectations, slope expectations) with 19 items. The mediating variable experience quality was adapted from Parasuraman, Zeithaml [43] scale (5 items). Visitor satisfaction was evaluated through Westbrook and Oliver [83] four-item scale, assessing overall satisfaction with skiing experiences. All items utilized a Likert five-point scale (1 = Strongly Disagree to 5 = Strongly Agree). The questionnaire comprised five sections: demographic characteristics (e.g., age, gender, skiing experience), sports tourism motivation, tourist expectation, experience quality, and satisfaction. This multidimensional measurement approach enabled comprehensive capture of latent variables among leisure ski tourists. To ensure the cultural adaptability of scales in the Chinese skiing context, two Chinese English professors performed independent back-translations. The Kappa coefficient (κ = 0.85) reached statistical significance.

After conducting confirmatory factor analysis (CFA), items with factor loadings below <  .05 were removed for discriminant validity purposes. Subsequently, items demonstrating significant modification indices that compromised model fit indices were iteratively deleted through re-specification. The psychology professor was invited to comprehensively justify both removed and retained measurement items, ensuring the scale’s psychometric suitability for assessing Chinese skiing enthusiasts’ psychology within the Chinese skiing context. The final measurement model retained 16 items, which are presented in Table 1.

**Table 1:** Measurement of latent variables.

Latent variable	Manifest variables	Measure description
Sport tourism motivation	Satisfaction with friendship	Average of 4 5-point scales^1^
	Satisfaction with solitude	Average of 4 5-point scales
	Satisfaction with challenge	Average of 4 5-point scales
	Satisfaction with intrinsic factor	Average of 3 5-point scales
	Satisfaction with achievement	Average of 3 5-point scales
	Satisfaction with nature	Average of 3 5-point scales
Tourist expectation	Expectation with ski resort conditions	Average of 7 5-point scales^2^
	Expectation with price and cost	Average of 4 5-point scales
	Expectation with services and supporting facilities	Average of 3 5-point scales
Tourist satisfaction	Sports tourism made me feel happy	−^3^
	I am satisfied with the energy of sports tourism	−
	Compared to previous travel experiences, sports tourism made me more satisfied	−
Experience quality	This sports tourism provided me with a high- quality travel experience	−^4^
	Compared to other travel experiences, this sports tourism experience was better	−
	The sports tourism experience is worth remembering	−
	The sports tourism experience made a lasting impression on me	−

^1^scored as 1 = strongly disagree, 5 = strongly agree; Items based on Seo, Lewin [81].

^2^scored as 1 = strongly disagree, 5 = strongly agree; Items based on Sun, Xie [82].

^3^scored as 1 = strongly disagree, 5 = strongly agree; Items based on Westbrook and Oliver [83].

^4^scored as 1 = strongly disagree, 5 = strongly agree; Items based on Parasuraman, Zeithaml [43].

### Common method variance

As with all self-reported data, there is a potential for common method biases resulting from multiple sources [84]. Therefore, we performed statistical analyses to assess the severity of common method bias. First, Harman’s single-factor test protocol [85], a principal component analysis without rotation was performed on all items. The results revealed a cumulative explained variance of 80.79%, with the largest eigenvalue accounting for 12.93% of the total variance. Since this proportion remained below the conventional 50% threshold, the analysis concluded no substantial CMV contamination was present. Second, according to Podsakoff and williams [84,86], and following Anderson [87] procedures for testing CMV, we included in the AMOS model a common method factor whose indicators included all the principal constructs’ indicators and calculated each indicator’s variances substantively explained by the principal construct and by the method. The results demonstrate that the average substantively explained variance of the indicators is  .752, while the average method-based variance is  .013. The ratio of substantive variance to method variance is about 58:1. This ratio far outperforms the 42:1 reference value recommended by previous studies [87]. In addition, most method factor loadings are not significant. Given the small magnitude and insignificance of method variance, we contend that the method is unlikely to be a serious concern for this study.

### Data analysis

Data analysis was conducted using IBM SPSS Statistics and AMOS 26.0. To test the research hypotheses, a two-step sequential approach involving measurement model development followed by structural equation modeling was implemented [88]. To minimize parameter estimation complexity, enhance numerical stability, and address potential violations of normality assumptions [89], we reduced the second-order measurement models for latent variables Sports Tourism Motivation and Tourist Expectation to first-order specifications following Little, Cunningham [90] recommendations. Subsequently, confirmatory factor analysis (CFA) was conducted on the four resulting measurement models.

The measurement model was tested using CFA with the maximum likelihood method [91]. Model fit evaluation employed multiple fit indices: χ²/df ratio, root mean square error of approximation (RMSEA), standardized root means square residual (SRMR), comparative fit index (CFI), goodness-of-fit index (GFI), adjusted goodness-of-fit index (AGFI), and Tucker-Lewis index (TLI) [92]. Criterion thresholds for acceptable model fit followed established guidelines: χ²/df < 5 [93], RMSEA < 0.08 [80], SRMR < 0.08, CFI and TLI > 0.95 [94], and GFI and AGFI > 0.90 [95].

The measurement model was systematically evaluated through multiple validity assessments: item reliability, construct reliability, convergent validity, and discriminant validity. Criterion thresholds strictly followed Hair, Black [96] recommendations for each indicator. Specifically, Item reliability was assessed using standardized factor loadings, with a minimum threshold of ≥0.50; Construct reliability was determined via composite reliability (CR), where values ≥0.70 denoted satisfactory internal consistency; Convergent validity was evaluated using average variance extracted (AVE), with ≥0.50 indicating adequate construct representation; Discriminant validity was tested by comparing squared inter-construct correlations with corresponding AVE values, requiring correlation coefficients < AVE for all construct pairs.

Following the identification of a satisfactory measurement model, structural equation modeling (SEM) analysis was conducted using the maximum likelihood method combined with Bollen-Stine bootstrap [97] to test the research hypotheses. The overall model fit evaluation maintained strict adherence to the identical fit criteria previously established for the measurement model

Regarding mediation analysis, we employed MacKinnon [98] bootstrap-based approach as an alternative to traditional causal step methodology and Sobel’s test. Following MacKinnon, Lockwood [99] product distribution theory, specific indirect effect estimates were computed using 5,000 bootstrap samples with 95% confidence intervals [98]. This methodological choice enhances statistical power and addresses potential issues with small sample distributions commonly encountered in mediation research.

## Results

### Reliability and validity of the scale

As shown in Table 2, all standardized factor loadings in the measurement models exceeded the critical threshold of >0.05. Composite reliabilities for all constructs were consistently above 0.90, indicating excellent construct reliability. The average variance extracted (AVE) values for each construct also surpassed the recommended threshold of >0.50, suggesting strong convergent validity. Model fit indices for the measurement models (Table 3) demonstrated satisfactory alignment with theoretical expectations. Furthermore, as evidenced by Table 4, the squared inter-construct correlations were systematically lower than their corresponding AVE values, providing robust evidence for discriminant validity across all construct pairs.

**Table 2:** Reliability and validity of the scale.

Dimension	Item	Unstd.	S.E.	*Z*	*p*	Std.	SMC	CR	AVE
STM	STM1	1.000				0.793	0.629	0.965	0.821
	STM2	1.084	0.057	19.038	***	0.908	0.824		
	STM3	1.089	0.057	19.204	***	0.913	0.834		
	STM4	1.057	0.053	19.943	***	0.936	0.876		
	STM5	1.138	0.057	20.067	***	0.940	0.884		
	STM6	1.090	0.055	19.992	***	0.938	0.880		
TE	TE1	1.000				0.984	0.968	0.966	0.906
	TE2	0.978	0.022	43.967	***	0.954	0.910		
	TE3	0.936	0.027	34.907	***	0.916	0.839		
TS	TS1	1.000				0.976	0.953	0.968	0.909
	TS2	0.994	0.025	39.701	***	0.945	0.893		
	TS3	0.999	0.026	38.180	***	0.938	0.880		
EQ	EQ1	1.000				0.919	0.845	0.967	0.881
	EQ2	1.048	0.034	31.150	***	0.950	0.903		
	EQ3	0.982	0.032	30.658	***	0.946	0.895		
	EQ4	0.979	0.033	29.732	***	0.938	0.880		

Note: STM- Sport tourism motivation, TE- Tourist expectation, TS-Tourist satisfaction, EQ- Experience quality.

**Table 3:** Measurement model fit.

Fit Indices	Measurement Model				Reference Value
	STM	TE (3 items)	EQ	TS (3 items)	
	Model Value				
χ²/df	1.894	−	0.766	−	< 5
RMSEA	0.053	−	0.049	−	< 0.08
SRMR	0.010	−		−	< 0.08
CFI	0.997	−	0.999	−	> 0.95
TLI	0.994	−	0.997	−	> 0.95
GFI	0.983	−	0.994	−	> 0.90
AGFI	0.960	−	0.971	−	> 0.90

Note: STM- Sport tourism motivation, TE- Tourist expectation, TS-Tourist satisfaction, EQ- Experience quality

**Table 4:** Discriminant validity.

Dimension	AVE	1	2	3	4
EQ	0.881	0.939			
TS	0.909	0.964	0.953		
TE	0.906	0.867	0.853	0.952	
STM	0.821	0.887	0.905	0.841	0.906

Note: STM- Sport tourism motivation, TE- Tourist expectation, TS-Tourist satisfaction, EQ- Experience quality

### Model hypothesis test

#### Direct effects test.

Following the Bollen-Stine bootstrap procedure, the final model fit indices were: χ²/df = 2.627, SRMR = 0.029, RMSEA = 0.074, GFI = 0.968, AGFI = 0.960, CFI = 0.980, and TLI = 0.975, indicating excellent model fit. As shown in Table 5, the analysis revealed significant positive correlations: Sport tourism motivation exhibited strong positive associations with tourist expectation (r = .86, p < .01) and experience quality (r = .60, p < .01), as well as tourist satisfaction (r = .26, p < .01). Furthermore, the standardized direct effects of sport tourism motivation on tourist expectation (β = .84, p < .01), experience quality (β = .54, p < .01), and tourist satisfaction (β = .23, p < .01) were all statistically significant. These results provide empirical support for Hypotheses H1, H2, and H3.

**Table 5:** Test results of path relationship hypothesis.

Hypothesis	Path relationship	Unstd.	S.E.	*Z*	*p*	Std.	Results
H1	STM → TS	0.257	0.055	4.683	***	0.233	S
H2	STM → TE	0.856	0.052	16.585	***	0.841	S
H3	STM → EQ	0.602	0.066	9.107	***	0.538	S
H4	EQ → TS	0.744	0.057	13.067	***	0.756	S
H5	TE → TS	0.001	0.048	0.025	0.980	0.001	N
H6	TE → EQ	0.455	0.059	7.687	***	0.414	S

Note: STM- Sport tourism motivation, TE- Tourist expectation, TS-Tourist satisfaction, EQ- Experience quality.

For Hypotheses H4 and H5, the analysis revealed that a strong positive association between experience quality and tourist satisfaction (r = .74, p < .01), with the standardized direct effect (β = .76, p < .01), while showing no significant association between tourist expectation and tourist satisfaction (r = .001, p > .05), whereas its direct effect on tourist satisfaction remained non-significant (β = .001, p > .05). These results provide support for Hypothesis H4 but reject Hypothesis H5.

The path analysis revealed that tourist expectation exhibited a strong positive correlation with experience quality (r = .46, p < .01), the standardized direct effect of tourist expectation on experience quality was statistically significant (β = .41, p < .01). The result provides robust empirical support for Hypothesis H6.

#### Mediation effect test.

To examine the indirect effects of the dependent variable through mediating variables, we conducted both percentile bootstrapping and bias-corrected percentile bootstrapping with 95% confidence intervals (5,000 bootstrap samples). Following MacKinnon, Lockwood [99] product distribution theory, we calculated specific indirect effects for each hypothesized mediating pathway. As shown in Table 6, the bootstrap analysis revealed: A significant positive mediating effect of Sport tourism motivation on tourist satisfaction through tourist expectation and experience quality (standardized indirect effect = .29 95%CI [0.12–0.55], [0.09–0.51]); A significant positive mediating effect of Sport tourism motivation on tourist satisfaction through experience quality alone (standardized indirect effect = .45 95%CI [0.16–0.90], [0.13–0.85]). However, the mediating effect through tourist expectation alone (standardized indirect effect = .001, 95% CI [−0.16–0.21], [−0.15–0.22]) failed to achieve statistical significance as its confidence interval included zero.

**Table 6:** Results of mediation effects.

Path relationship	Point Estimate			Bootstrap Test					
		Coefficient		Bias-corrected Percentile			Percentile		
		SE	*Z*	Lower	Upper	p	Lower	Upper	P
Specific indirect effect									
STM → TE → EQ → TS (1)	0.290	0.105	2.762	0.119	0.553	0.001	0.093	0.506	0.002
STM → TE → TS (2)	0.001	0.098	0.010	−0.160	0.205	0.953	−0.151	0.219	0.939
STM → EQ → TS (3)	0.448	0.187	2.396	0.156	0.897	0.001	0.130	0.853	0.001
Direct effect									
STM → TS	0.257	0.143	1.797	0.053	0.625	0.006	0.035	0.587	0.011
Total effect	0.997	0.107	9.318	0.808	1.227	0.001	0.813	1.244	0.000
Comparison of mediating paths									
(1) vs. (2)	0.289	0.144	2.007	0.047	0.637	0.021	−0.013	0.560	0.061
(1) vs. (3)	−0.158	0.253	−0.625	−0.683	0.293	0.518	−0.688	0.292	0.513
(2) vs. (3)	−0.447	0.257	−1.739	−0.991	−0.006	0.046	−0.952	0.021	0.065

Note: STM- Sport tourism motivation, TE- Tourist expectation, TS-Tourist satisfaction, EQ- Experience quality.

Both Bias-Corrected and Percentile bootstrapping methods consistently showed non-overlapping confidence intervals for the first two pathways (1 vs. 2 and 1 vs. 3), while no significant differences were observed between Pathways 2 and 3 using either method. Based on methodological precedence, we prioritized the Bias-Corrected Percentile results for interpretation due to it has the highest statistical power [100]. These findings provide support for Hypotheses H7 and H9, while rejecting Hypothesis H8. The differential mediation effects suggest that experience quality plays a more critical role in mediating the relationship between Sport tourism motivation and satisfaction compared to tourist expectation alone.

## Discussion

Leisure tourism skiing constitutes one of the most significant sectors within the global sports tourism industry. This study takes Changbaishan Wanda International Ski Resort, a renowned ski destination in China, as the research context and targets leisure skiing tourists as the subject of investigation. Employing structural equation modeling (SEM), we establish a theoretical framework incorporating four key variables—sports tourism motivation, tourist satisfaction, tourist expectation, and experience quality—to systematically examine interrelationships among these constructs and elucidate the underlying mechanisms driving tourist satisfaction in the leisure skiing sector.

The findings reveal that sports tourism motivation exerts a significant positive influence on tourist satisfaction, tourist expectation and experience quality. Tourist expectations demonstrate a robust positive influence on perceived experience quality, yet show no significant direct association with overall satisfaction. Perceived experience quality emerges as a critical determinant of tourist satisfaction. Notably, tourist expectation and experience quality collectively mediate the relationship between sports tourism motivation and satisfaction through two distinct pathways: an independent mediation effect and a sequential mediation effect. However, the independent mediation effect via expectation alone exhibits statistical insignificance. Bootstrapping analysis using Bias-Corrected Percentile methods further corroborates that the sequential mediation pathway and the independent mediation effect of experience quality significantly outperform the independent mediation effect of expectation in explaining satisfaction variance.

First, the findings of this study demonstrate that sports tourism motivation exerts significant positive direct effects on leisure skiing tourist satisfaction, tourist expectation and perceived experience quality. Tourist satisfaction, a cornerstone construct in sports tourism research, serves as a critical focal point for theoretical and practical exploration. Firstly, the finding that sports tourism motivation positively influences tourist satisfaction is corroborated by existing empirical evidence. Deng, Wang [7] revealed in their investigation of China’s themed sports tourism routes that tourism motivation not only directly affects satisfaction but also indirectly enhances loyalty to sports-themed tourism through satisfaction as a mediator. This aligns with China’s emerging leisure skiing boom, an industry invigorated by governmental policy initiatives following the 2022 Beijing Winter Olympics. The national campaign advocating for 300 million citizens to engage in winter sports, coupled with substantial policy support for winter tourism participation, has significantly amplified public enthusiasm for leisure skiing. Consequently, the synergistic interaction of intrinsic and extrinsic motivations among leisure skiers collectively contributes to elevated satisfaction levels. Respondents in this study, evidently influenced by this broader contextual backdrop, exhibited significant enthusiasm for leisure skiing. Additionally, on-site surveys revealed that the demographic profile of leisure skiing participants skewed heavily toward individuals from northern China. Given northern China’s prolonged winter season, outdoor ice and snow sports represent the most common form of physical activity for northerners—a factor that indirectly explains why the motivation driving respondents’ engagement in leisure skiing has a pronounced positive direct effect on their satisfaction. Secondly, Lee, Jeon [70] observed a positive correlation between Chinese tourists’ pre-travel motivations and their expectations during South Korean vacations—a finding partially supportive of the current results. Their research suggests that Chinese tourists entering cultural or service-oriented contexts anticipate meeting predefined thresholds of satisfaction, a phenomenon analogous to leisure skiers’ heightened expectations for facility quality, accommodation services, equipment provisions, and entertainment offerings prior to experiencing ski resorts. Such psychological predispositions logically stem from strong motivational drivers. In fact, the influence of motivation on expectation is not unidirectional. Hsu, Cai [47] study on Chinese outbound tourists, for instance, found that expectations regarding the purpose of outbound travel directly shape motivation to visit the destination. This explains why respondents exhibit such strong motivation and high expectations toward leisure skiing—it stems from the added value of leisure skiing activities, which may surpass that of skiing itself. Thirdly, this study identifies a significant positive relationship between sports tourism motivation and perceived experience quality. Deng, Wang [7] similarly reported that motivation not only directly impacts experience quality but also mediates its effect on behavioral loyalty. This perspective is theoretically grounded in Baker and Crompton [101] assertion that highly motivated tourists engage more deeply with activities, thereby generating superior experiential outcomes. Further supporting this notion, Joyner, Kline [102] posited that motivation represents tourists’ psychological needs and desires toward destinations and activities, which predispose them to develop favorable affective orientations toward their travel experiences.

Second, this study identifies a significant positive relationship between tourist expectations and perceived experience quality, with the latter demonstrating a similarly robust influence on tourist satisfaction. Theoretically, when leisure skiers perceive high-quality services across destination attributes—including facility infrastructure, staff professionalism, accommodation standards, and culinary offerings—their post-experience satisfaction aligns with rational expectations. This logic finds empirical support in Deng, Wang [7], whose mediation analysis of sports tourism confirmed that tourist expectations directly enhance satisfaction within leisure-oriented contexts. However, our unexpected finding reveals that tourist expectations exert no direct significant effect on satisfaction in the current model. This outcome may stem from cognitive dissonance generated when high-expectation tourists encounter discrepancies between anticipated and actual experiences during leisure skiing. Such dissonance could suppress the direct satisfaction pathway, thereby reinforcing experience quality as a full mediator between expectations and satisfaction—a configuration observed in partial mediation models. Lee, Jeon [70] similarly validated this mediating mechanism in their study of Chinese tourists’ loyalty in South Korea, where expectations influenced satisfaction exclusively through experiential quality. A critical divergence emerges, however, in the relationship between expectations and experience quality. Our results indicate that leisure skiers’ pre-trip motivations significantly enhance their in-situ experience quality, contrasting with Lee, Jeon [70] observation that cross-cultural tourism expectations (e.g., China-to-Korea travel) exhibit heightened sensitivity and fragility due to cultural friction. We attribute this discrepancy to contextual factors including geographical settings and cultural environments, which moderate how expectations translate into experiential outcomes. Specifically, the homogeneity of domestic winter sports culture in China may mitigate potential expectation-reality gaps compared to international tourism scenarios involving cultural adaptation.

Third, this study identifies tourist expectations and perceived experience quality as pivotal determinants influencing leisure skiing satisfaction, with experience quality serving as a critical mediator between motivation and satisfaction. The findings demonstrate that, compared to pre-trip expectations, in-situ experiential quality holds greater substantive significance. Consequently, winter sports destinations may enhance satisfaction and revisit intention through strategic optimization across three dimensions: skiing infrastructure, accommodation-culinary systems, and entertainment services. For instance, in terms of skiing infrastructure, implementing scientific slope grading systems, real-time snow maintenance protocols, tiered coaching programs, and intelligent safety equipment, supplemented by themed activities (e.g., night lighting shows, cultural check-in points); accommodation-culinary systems: offering diversified lodging options, specialty gastronomy programs, and health management services; Entertainment services: developing post-skiing experiences such as thermal therapy zones and family-oriented cultural activities. This enhancement should be operationalized through dynamic pricing algorithms and scenario-based staff training to establish closed-loop service ecosystems, ultimately achieving synergistic improvements in satisfaction and loyalty. Secondly, our most novel contribution reveals that tourist expectations and experience quality collectively mediate motivation-satisfaction relationships via sequential mediation mechanisms. This further elucidates why the total effect size of motivation on satisfaction in this study surpasses prior research findings. For example, Deng [7] observed in their study on tourism motivation and tourist loyalty that the effect size of tourism motivation on tourist satisfaction was merely 0.440 (medium)—considerably smaller than the 0.997 (large, Cohen’s d 0.2 small, 0.5 medium, 0.8 large [103]) reported here. Comparative mediation analysis demonstrates that the sequential pathway and experience quality’s direct mediation effect significantly outperform isolated expectation-mediated pathways. This underscores experience quality’s pivotal role as a mediator—consistent with Baker and Crompton [101] conceptualization of satisfaction as a post-experience emotional state reflecting fulfillment.

## Implications

First, this study advances theoretical understanding of leisure skiing consumption behavior by unveiling the sequential mediation mechanism through which tourism motivation influences tourist expectations, perceived experience quality, and satisfaction. We confirm that tourism motivation—acting as an antecedent variable—amplifies satisfaction via this sequential pathway. These findings enrich scholarly insights into how motivation shapes satisfaction within the leisure skiing domain. For instance, compared to prior work—including Tourist Behavioral Model [47] and stimulus-response model linking tourism motivation to tourist loyalty [7]—this study identifies tourist expectations and perceived experience quality as critical mediating factors that strengthen tourist satisfaction. As a core component of leisure skiing tourism, leisure experience is an indispensable element that cannot be neglected in skiing tourism. Consequently, leisure experience must align with consumers’ expectations and address their practical needs. From this perspective, leisure skiing destinations as platforms for delivering tourists’ leisure experiences; accordingly, our results offer actionable managerial insights for destination practitioners.

Second, at the practical level, leveraging tourist expectations and perceived experience quality—two key mediating factors—in the actual leisure skiing experience is critical for enhancing tourist satisfaction. Based on the magnitude of their mediation effects, motivation exerts the strongest indirect effect on satisfaction via perceived experience quality, so managers of leisure skiing tourism destinations should first focus on enhancing tourists perceived experience quality, which includes but is not limited to safety assurance, facility maintenance, entertainment engagement, and instruction professionalism. This suggests that ski tourism destinations need to establish a management system centered on tourists perceived experience quality as a core parameter to optimize resource allocation and quality monitoring for visitor experiences. Additionally, the serial mediation effect of motivation on satisfaction through tourist expectations and perceived experience quality is the next strongest, making tourist expectations another non-negligible factor, which prompts the development of a precise dynamic tourist demand identification system to align tourist expectations with actual service delivery. For example, implementing tiered certification systems and coach accreditation programs for skill-seeking tourists can strengthen their expectations for professional services, while prioritizing community engagement activities for social tourists helps sustain their participation enthusiasm. This motivation-expectation-perceived experience-satisfaction chain paradigm provides a theoretical basis for narrowing experience gaps and optimizing resources, thereby facilitating the transition of ski tourism destinations from extensive service models to precision supply chain models.

### Limitations and future research

While this study focuses on Changbaishan Wanda International Ski Resort in Jilin Province as the primary research site—adequately reflecting operational characteristics of high-end ski destinations—it inherently possesses geographical representativeness limitations. The regional selectivity may restrict the generalizability of findings to China’s diverse skiing markets, which exhibit variations in climatic conditions, tourist demographics, and consumption hierarchies. For instance, our field survey revealed that the majority of tourists in the study area originate from northern China, a region where winter skiing constitutes an integral component of local daily culture and lifestyle. This contextual characteristic may exert an impact on the assessment of tourist motivations. Future research should establish a multi-regional stratified sampling framework to systematically compare established northern resorts, emerging southern resorts, and characteristic central-western resorts. This approach would enable analysis of spatial positioning characteristics in relation to patron demographics. Additionally, longitudinal tracking across seasonal cycles (peak, off-peak, and low seasons) could capture dynamic behavioral variations, thereby enhancing the explanatory power of conclusions under heterogeneous market environments.

This study employed convenience sampling methods reliant on voluntary participation from on-site visitors, potentially introducing self-selection bias and temporal-spatial coverage limitations. For instance, tourists actively completing surveys may exhibit stronger subjective expression tendencies (e.g., pronounced satisfaction or dissatisfaction inclinations), while the “silent majority” remained underrepresented. Furthermore, the concentration of data collection during peak winter seasons may overlook behavioral patterns of off-season visitors (e.g., summer skiing trainees), resulting in sample structures misrepresenting population characteristics. Future research should implement hybrid sampling strategies combining fixed exit-point questionnaires for continuous temporal coverage with supplemental digital surveys via online platforms. Stratified random sampling techniques incorporating visitor attributes (age, skiing proficiency, expenditure levels) through quota allocation would enhance representativeness. For specialized cohorts such as youth education groups and corporate retreats, purposive sampling combined with in-depth interviews could address quantitative research limitations, integrating qualitative insights to improve explanatory depth and methodological robustness.

Last but not least, the conceptual model formulated through literature review adopts a linear mediation framework grounded in hypotheses, characterized by unidirectional causal relationships. This design may inadvertently obscure the influence of additional factors on the motivation-satisfaction pathway. Owing to limitations imposed by research hypotheses and article length, this study did not adequately examine other potentially relevant variables. We recommend that future research build on this foundation to explore alternative factors or models that could modulate the mediating mechanisms linking motivation to satisfaction.

## Conclusion

This study employs structural equation modeling to investigate the interrelationships among four key constructs: sports tourism motivation, tourist satisfaction, tourist expectation, and experience quality. The analytical framework aims to systematically test hypothesized pathways and elucidate the driving mechanisms underlying satisfaction enhancement in leisure skiing tourism. The results reveal a significant sequential mediation effect wherein tourist expectation and perceived experience quality mediates the relationship between sports tourism motivation and satisfaction. Notably, the direct mediation effect of experience quality demonstrates substantially greater explanatory power compared to the standalone mediation effect of tourist expectation. These findings provide critical theoretical and practical implications for optimizing service delivery systems and designing targeted interventions in leisure skiing destinations.

## Supporting information

S1 Data Sample Covariances. (XLSX)

## Abbreviations

CFA: Confirmatory factor analysis
SEM: Structural equation modeling
STM: Sport tourism motivation
TE: Tourist expectation
TS: Tourist satisfaction
EQ: Experience quality
